# Rare *CYLD* Variants in Chinese Patients With Amyotrophic Lateral Sclerosis

**DOI:** 10.3389/fgene.2021.740052

**Published:** 2021-11-12

**Authors:** Xiaojing Gu, Yongping Chen, Qianqian Wei, Yanbing Hou, Bei Cao, Lingyu Zhang, Ruwei Ou, Junyu Lin, Kuncheng Liu, Bi Zhao, Huifang Shang

**Affiliations:** Department of Neurology, Laboratory of Neurodegenerative Disorders, Rare Disease Center, West China Hospital, Sichuan University, Chengdu, China

**Keywords:** CYLD lysine 63 deubiquitinase gene, amyotrophic lateral sclerosis, mutation screening, phenotype, burden analysis

## Abstract

**Background:** CYLD Lysine 63 Deubiquitinase gene (*CYLD*) was recently identified to be a novel causative gene for frontal temporal dementia (FTD)-amyotrophic lateral sclerosis (ALS). In the current study, we aimed to (1) systematically screen the mutations of *CYLD* in a large cohort of Chinese ALS patients, (2) study the genotype–phenotype correlation, and (3) explore the role of *CYLD* in ALS *via* rare variants burden analysis.

**Methods:** A total of 978 Chinese sporadic ALS (sALS) patients and 46 familial ALS (fALS) patients were sequenced with whole-exome sequencing and analyzed rare variants in *CYLD* with minor allele frequency <0.1%.

**Results:** In total, seven rare missense variants in *CYLD* have been identified in 7 (0.72%) patients among 978 sALS patients. Two (4.3%) rare missense variants were identified among the 46 fALS cases, in which one patient was diagnosed as having comorbidity of ALS and progressive supranuclear palsy (PSP). Moreover, the burden analysis indicated no enrichment of rare variants in *CYLD* among patients with ALS.

**Conclusion:** In conclusion, our study extended the genotype and phenotype of *CYLD* in ALS, but the pathogenicity of these variants needs to be further verified. Moreover, burden analysis argued against the role of *CYLD* in the pathogenesis of ALS. More studies from different ethnicities would be needed.

## Introduction

Recently, a heterozygous missense variant in CYLD Lysine 63 Deubiquitinase gene (*CYLD*) was found to co-segregate with the FTD-ALS in an autosomal dominant inherited European Australian family (Dobson-stone et al., 2020). Further functional studies revealed that mutated CYLD can lead to an increased lysine 63 deubiquitinase activity and gave rise to the pathogenesis of ALS and FTD (Dobson-stone et al., 2020). A subsequent study on a cohort of 65 Portuguese FTD patients identified two rare *CYLD* variants in 2 FTD patients (Tábuas-Pereira et al., 2020). ALS and FTD are considered as two related diseases in a continuous clinical spectrum that share not only clinical manifestations, but also genetic features and neuropathological abnormalilities (Murphy et al., 2007), while differences in genetic background have been described among ALS patients from different ethnicities (Wei et al., 2017). Therefore, the current study aimed to study the role of *CYLD* in Chinese patients with ALS.

## Methods

We recruited 978 Chinese sporadic ALS (sALS) patients and 46 familial ALS (fALS) probands admitted to the Department of Neurology, West China Hospital of Sichuan University. All the ALS patients were diagnosed based on the El Escorial revised criteria for definite or probable ALS (Brooks et al., 2000). Written and signed informed consent were obtained from all the participants. The study was approved by the Ethics Committee of West China Hospital, Sichuan University.

Genomic DNA was collected from peripheral blood leukocytes and underwent whole-exome sequencing (WES). Procedures of WES and variants annotation were performed as previously described (Gu et al., 2020). *C9orf72* was sequenced using Repeat-primed PCR and gel electrophoresis methods as previously described (Chen et al., 2015). Patients with mutations in known ALS causative genes such as *SOD1*, *FUS*, *C9orf72, TARDBP*, and *TBK1* were excluded (Gregory et al., 2020). The average depth for *CYLD* was over 100×. Considering the autosomal dominant-inherited model for *CYLD*, we selected the variants according to the minor allele frequency (MAF) less than 0.1% in public databases, and the pathogenicity was based on multiple lines of *in silico* prediction tools including SIFT, Polyphen2, LRT, and Mutation Taster. Sanger sequencing was performed to confirm the variants.

Regarding burden analysis, controls were from the gnomAD East Asian population non-neuro dataset (https://gnomad.broadinstitute.org/). Combined Annotation Dependent Depletion (CADD) integrates predictions from numerous bioinformatic algorithms into a single “C-score” and ranks all possible nucleotide changes in the genome based on potential to disrupt gene/protein function (Uitterlinden et al., 2017). In accordance with the previous study, we defined a stringent CADD C-score ≥12.37 as likely damaging variants, representing the top 2% most damaging of all possible nucleotide changes in the genome—this subset is enriched for known pathogenic alleles (Uitterlinden et al., 2018). Moreover, the in-frame deletion/insertion variants lead to the change of the protein length and are classified as moderately strong evidence for pathogenicity (Richards et al., 2015). Therefore, in-frame deletion/insertion variants were also considered as damaging variants in the current study. Five different algorithms, including Comprehensive Approach to Analyzing Rare Genetic Variants (CARV), Sum of Squared Score (SSU), Sum Test (SUM), Cumulative Minor Allele Test (CMAT), and Bayesian Score Test (BST) were used for burden analysis with AssotesteR Package in the rare variants level and rare deleterious variants level (Lee et al., 2014).

## Results

**FIGURE 1:**
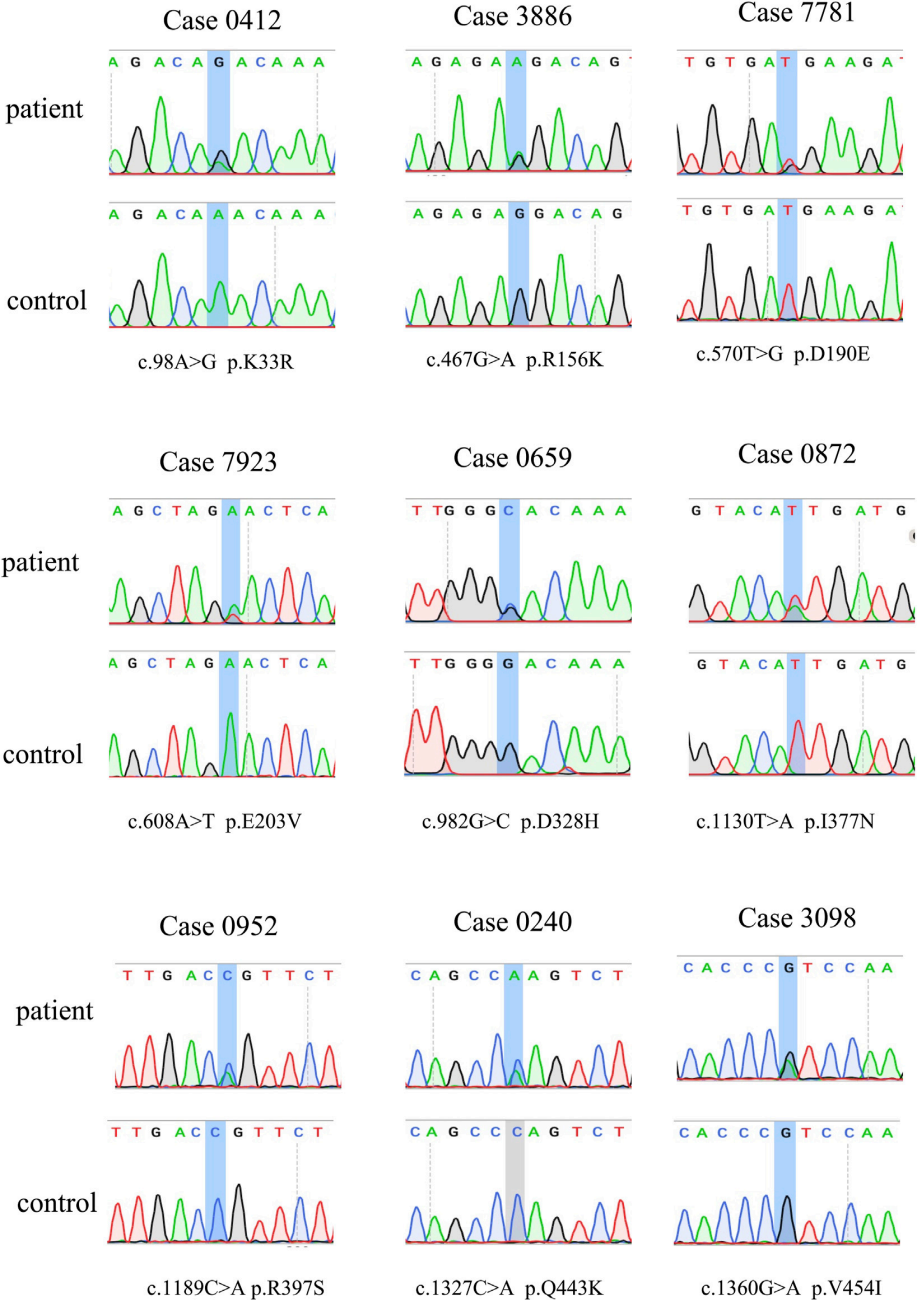
Sanger sequencing for the observed variants in *CYLD*.

The demographic characteristics of all patients are listed in Supplementary Table S1. In total, seven rare missense variants (p. K33R and p. R156K in the exon 4, p. E203V in the exon 5, p. D328H in the exon 8, p. I377N in the exon 9, and p. R397S and p. V454I in the exon 10) in CYLD were identified in 7 (0.72%) patients from 978 sALS patients, and two (4.3%) rare missense variants (p. D190E in the exon 4 and p. Q443K in the exon 10) were identified in 2 patients from the 46 fALS cases (Table 1 and Figure 1). All the nine variants had a very low frequency or were even absent in the public databases of East Asia controls (PM2), and multiple lines of *in silico* prediction tools supported a deleterious effect (PP3). Therefore, all of them were classified into variants of uncertain significance (VUS) (Richards et al., 2015). No variant was found in the deubiquitinase domain of CYLD (amino acids 593–948), which was revealed to have a significant enrichment in FTD cases by the previous study (Dobson-stone et al., 2020).

**TABLE 1:** Detailed description of the observed rare variants in *CYLD*.

Case No.	Exon	c.DNA	Amino acid change	Depth	Alt reads	dbSNP	ExAC East Asia	gnomAD East Asia	SIFT	Poly phen2	LRT	Mutation taster	ACMG classification
0412	4	c.98A > G	p.K33R	157	88	rs565310513	0.000116	0.000173	T	B	D	D	VUS
3886	4	c.467G > A	p.R156K	155	77	—	—	—	T	D	D	D	VUS
7781	5	c.570T > G	p.D190E	90	43	—	—	—	T	B	N	D	VUS
7923	5	c.608A > T	p.E203V	154	71	—	—	—	D	D	D	D	VUS
0659	8	c.982G > C	p.D328H	37	24	—	—	—	D	P	D	D	VUS
0872	9	c.1130T > A	p.I377N	63	26	—	—	—	D	B	D	D	VUS
0952	10	c.1189C > A	p.R397S	98	48	rs149427272	0.000348	0.000231	T	B	N	D	VUS
0240	10	c.1327C > A	p.Q443K	203	100	rs764952788	0.000348	0.000289	T	P	D	D	VUS
3098	10	c.1360G > A	p.V454I	94	42	rs200451975	0.000116	0.000058	T	T	N	D	VUS

SIFT:D: deleterious (0–0.05), T: tolerated (>0.05).

PolyPhen2: D: Probably damaging (≥0.909), P: possibly damaging (0.447 ≤ pp2_hvar ≤ 0.909), B: benign (pp2_hvar ≤ 0.446).

LRT: likelihood ratio test; D: Deleterious; N: Neutral (identification of deleterious mutations within three human genomes).

Mutation Taster: D: disease causing; N: polymorphism; P: polymorphism automatic.

VUS: variant of uncertain significance.

The clinical features of the nine patients carrying rare variants in CYLD are listed in Supplementary Table S2. Most of the patients (8/9) had limb onset. The disease progression and survival time varied greatly among patients, but most of the patients were likely to have a relatively slow progression and long survival time. Two patients from six patients whose cognition were detailed assessed showed global cognitive impairment and frontal lobe dysfunction but did not present the typical features of FTD such as changes in behavior, personality, and aphasia.

Interestingly, among the nine patients, case 0659 had atypical ALS. He developed rigidity, bradykinesia, and weakness in both upper limbs at the age of 63. Then, he developed dysarthria 3 months later, and developed rigidity and weakness of both lower limbs 6 months later. He was diagnosed as having progressive supranuclear palsy (PSP) at the local hospital and was treated with levodopa but had no response. At the disease duration of 1 year since onset, he came to our clinic and was found to have difficulty in shifting his gaze horizontally and vertically, muscle atrophy of both hands, and increased muscle tone of limbs. Muscle strength of the proximal and distal part of lower limbs was grade 4^+^ and grade 3^+^, respectively, whereas muscle strength of lower limbs was grade 5. Pyramidal signs were found. The electromyographic examination indicated diffused neurogenic degeneration in the cervical, thoracic, and lumbosacral spinal cord segments. Therefore, the patient was diagnosed to have a comorbidity of ALS and PSP. The patient had a faster progression, and the muscle strength of extremities decreased to grade 2 after 3 months. The patient died of respiratory failure at the disease duration at 20.8 months. The patient’s mother developed cognitive impairment at the age of 74, progressed to have muscle weakness, and died within 2 years (Supplementary Figure S1). Moreover, two younger brothers of the proband’s mother both developed muscle weakness around 50 years old and died within several years since onset. Backtracking the previous generation, the patient’s grandmother on his mother’s side was recalled having dementia; however, the detailed medical history could not be obtained. Unfortunately, all the affected patients in the pedigree had died, so the co-segregation of the variant was not available.

To further evaluate the accumulated association of the rare variants in *CYLD* with ALS, we did gene-level rare variant burden analyses. There was no significant enrichment of putative pathogenic variants in our study when compared with the gnomAD East Asian control (Supplementary Table S3). Furthermore, when the variants were divided into “damaging” and “benign” based on the cutoff value at 12.37 of CADD scores, there were also no differences between patients and controls (Supplementary Table S3).

## Discussion

CYLD is a deubiquitinating enzyme that targets Lys63-linked ubiquitin chains and regulates biological pathways such as the NF-ĸB signaling pathway and autophagy (Kovalenko et al., 2003; Yamashita et al., 2019). Loss-of-function mutations in *CYLD* have been shown to cause skin diseases, while very recently, missense mutations in *CYLD* were found to cause ALS-FTD *via* gain-of-function mechanism (Dobson-stone et al., 2020).

In the current study, nine patients were found to carry rare missense variants in *CYLD* with the MAF <0.1%. In total, seven rare missense variants in *CYLD* have been identified in 7 (0.72%) patients among 978 sALS patients, and two (4.3%) rare missense variants were identified among the 46 fALS cases, where familial cases had a relatively higher variation frequency. This phenomenon could be due to the following: firstly, as found in our previous study, familial cases are much more likely be caused by genetic causes than sporadic cases (40.6% in fALS vs. 8.6% in sALS) (Chen et al., 2021); secondly, the sample size of familial cases was relatively small. It is interesting to note that, besides the classical phenotype of ALS and ALS with cognitive impairment, our current study found one familial case presenting with a comorbidity of ALS and PSP who had rapid progression and short survival. The previous study on the large ALS-FTD pedigree reported that one patient had clinical diagnosis of FTD-ALS, Paget’s disease, and parkinsonism (Dobson-stone et al., 2020), whose pathological findings met neuropathological criteria for both cortical basal degeneration (CBD) FTLD with tau pathology (CBD FTLD-tau) and FTLD with type B TDP-43 pathology (type B FTLD-TDP). Moreover, TDP-43 pathology has also been reported in PSP (Koga et al., 2017). Therefore, our study further expanded the phenotype for *CYLD* mutation carriers. Moreover, two patients in our cohort (Patient 3886 and Patient 0952) had global cognitive impairments, frontal lobe dysfunction, and irritative symptoms but without FTD symptoms including behavior changes and aphasia.

**FIGURE 2:**
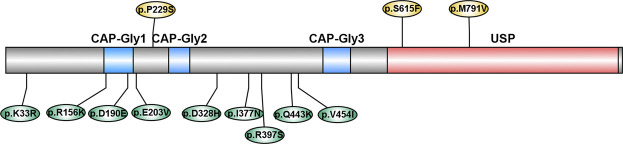
Schematic diagram of variants in *CYLD*. CAP-Gly: cytoskeleton-associated protein-glycine conserved domain; USP: ubiquitin-specific protease domain. Variants reported by previous studies were labeled above, and variants reported in our study were labeled below.

CYLD is composed of three N-terminal cytoskeleton-associated protein-glycine conserved domains (CAP-Gly), which are responsible for binding to microtubules and other proteins, and a C-terminal ubiquitin-specific protease domain (USP), which is functioning for the disassembly of Lys63-linked ubiquitin polymers (Komander et al., 2008). Most of the variants identified in our study were not located in the functional domain, while two variants (p. D190E and p. E203V) were located in the CAP-Gly domain. However, two variants reported by previous studies (p. S615F and p. M791V) were located in the USP domain (Dobson-stone et al., 2020; Tábuas-Pereira et al., 2020) (Figure 2). This evidence emphasized the genetic heterogeneity among different ethnicities.

For the rare variants burden analysis, we did not find a significant enrichment of rare variants of *CYLD* in Chinese patients with ALS, which was consistent with the finding of the original study, which only detected a significant enrichment of rare variants in the deubiquitinase domain of *CYLD* (amino acids 593–948) in FTD patients, but not in ALS either in the whole gene level or the deubiquitinase domain (Dobson-stone et al., 2020). These results indicated that rare variants in the deubiquitinase domain of *CYLD* may contribute to the risk for FTD but not ALS.

However, some limitations of our study should be acknowledged. First, the sample size of familial cases was relatively small and the pedigree validation of the familial case was not available, which compromised the evidence to support pathogenicity of these variants. Second, there are genetic heterogeneity across races and rare variants can be (sub) population specific; therefore, using public databases as control for burden analysis will cause some unavoidable bias. Although we used the East Asian population in gnomAD as our control, which could decrease the heterogeneity to some extent, it would be better to enroll controls from the same region. Thirdly, no functional study has been performed to confirm the pathogenicity of CYLD variants.

## Conclusion

In conclusion, the current study on a large cohort of Chinese patients with ALS suggested that the frequency of variant in *CYLD* is relatively uncommon in Chinese patients with sALS (0.72%) and fALS (4.3%), while the pathogenicity of the variants needs to be further verified. Our findings of ALS-PSP phenotype further expanded the phenotypic spectrum of the *CYLD* in ALS. Moreover, burden analysis argued against the role of *CYLD* for the risk of ALS. More studies from different ethnicities are needed.

## Data Availability

The datasets generated during and/or analyzed during the current study are available from the corresponding author on reasonable request.
