# Supplementary material for: Rare CYLD Variants in Chinese Patients With Amyotrophic Lateral Sclerosis
Source: Front Genet. 2021 Nov 12;12:740052. doi: 10.3389/fgene.2021.740052 (PMC8633398; doi:10.3389/fgene.2021.740052)
Supplement: Supplementary file 1 [file DataSheet1.PDF]

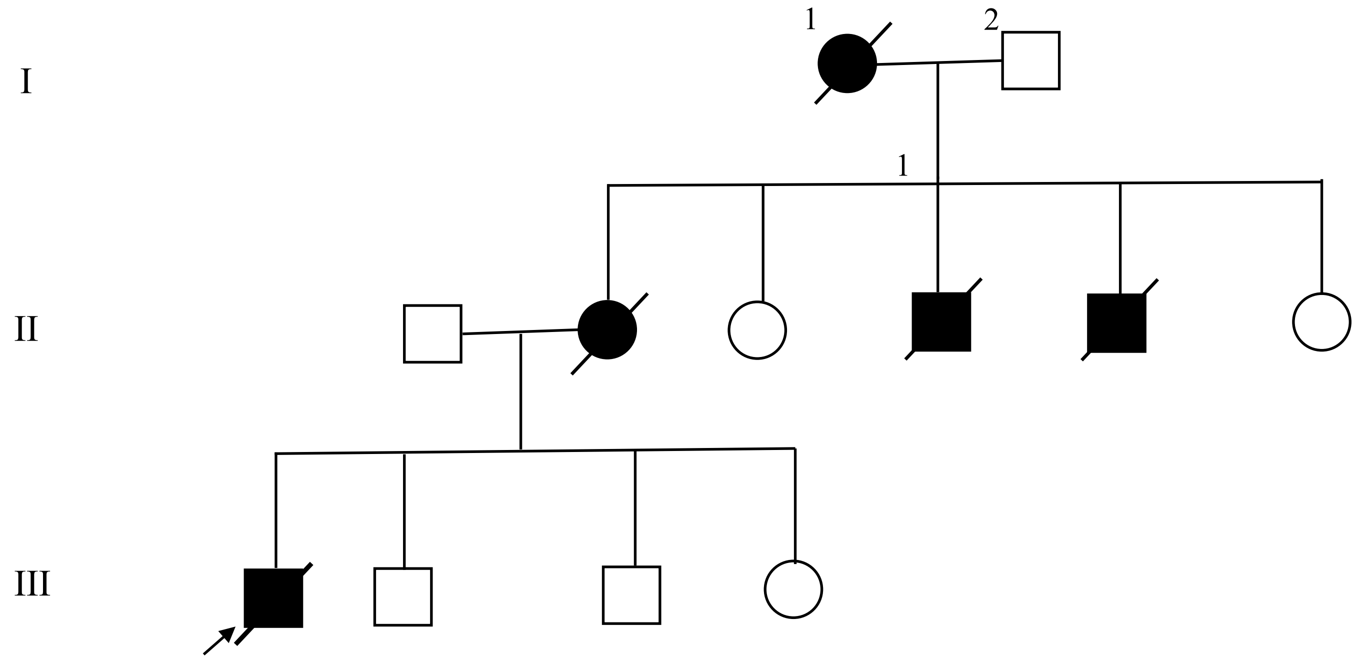

Supplementary figure 1: Pedigree the fALS case carrying variant in *CYLD*. Filled and empty symbols indicate individuals affected with ALS and without ALS, respectively.
