# Supplementary material for: Rare CYLD Variants in Chinese Patients With Amyotrophic Lateral Sclerosis
Source: Front Genet. 2021 Nov 12;12:740052. doi: 10.3389/fgene.2021.740052 (PMC8633398; doi:10.3389/fgene.2021.740052)
Supplement: Supplementary file 2 [file DataSheet2.DOCX]

**Supplementary Table 1. Demographic characteristics of all participants.**

| Variable | sALS | fALS |
| --- | --- | --- |
| Cases, N | 978 | 46 |
| Sex, M/F | 574/404 | 26/20 |
| Mean onset age (±SD, years) | 53.1±12.4 | 46.7±14.9 |
| Disease duration (±SD, months) | 17.7±17.4 | 27.6±44.6 |

**Supplementary Table 2. Clinical features of sALS patients carrying variants in *CYLD.***

| Case No | AAO  (years) | Disease duration | diagnostic certainty | Family  history | Site of onset | ALSFRS | Progression  (score/month) | Survival time  (months) | MOCA | ACER | FAB | FBI | FTD-Q | HAMD | HAMA |
| --- | --- | --- | --- | --- | --- | --- | --- | --- | --- | --- | --- | --- | --- | --- | --- |
| 0412 | 47.0 | 7.6 | Definite | - | LL | 18 | 3.95 | 9.6 | NA | NA | NA | NA | NA | NA | NA |
| 3886 | 48.1 | 7.2 | Possible | - | UL | 47 | 0.14 | 20.0(alive) | 20 | 64 | 17 | 5 | 29 | NA | 11 |
| 7781 | 44.0 | 12.6 | Definite | - | UL | 40 | 0.63 | 84.1(alive) | 29 | 99 | 18 | NA | NA | 8 | 0 |
| 7923 | 49.7 | 9.3 | Definite | + | Bulbar | 40 | 0.86 | 36.6 | NA | NA | NA | NA | NA | NA | NA |
| 0659 | 63.4 | 12.3 | Definite | + | UL | 25 | 1.87 | 26.2(alive) | NA | NA | NA | NA | 34 | NA | NA |
| 0872 | 59.2 | 13.3 | Definite | - | UL | 32 | 1.20 | 17.2 | 23 | NA | 15 | 3 | NA | 21 | 15 |
| 0952 | 55.1 | 5.3 | Probable | - | LL | 41 | 1.32 | 6.6 | 14 | 53 | 11 | 9 |  | 14 | 13 |
| 0240 | 53.4 | 5.1 | Definite | - | UL | 45 | 0.59 | 42.2(alive) | 28 | 88 | 18 | 0 | NA | 26 | 12 |
| 3098 | 49.0 | 14.2 | Possible | - | UL | 47 | 0.07 | 21.9(alive) | 25 | 84 | 18 | NA | NA | 2 | 0 |

UL: Upper limb; LL: Lower limb.

B-Bulbar; C-Cervical; T: Thoracic; L: Lumbosacral.

NA: not available.

**Supplementary table 3. Results for burden analysis.**

|  | p value of rare variants burden analysis | p value of rare damaging variants |
| --- | --- | --- |
| CARV | 0.838 | 0.670 |
| SSU | 0.838 | 0.670 |
| SUM | 0.328 | 0.270 |
| CMAT | 0.838 | 0.670 |
| BST | 0.838 | 0.670 |

CARV :Comprehensive Approach to Analyzing Rare Genetic Variants; SSU: Sum of Squared Score;SUM: Sum Test (SUM); CMAT: Cumulative Minor Allele Test; BST:Bayesian Score Test.
